# Effects of *Chlorella vulgaris* as a Feed Ingredient on the Quality and Nutritional Value of Weaned Piglets’ Meat

**DOI:** 10.3390/foods10061155

**Published:** 2021-05-21

**Authors:** Cátia F. Martins, José M. Pestana, Cristina M. Alfaia, Mónica Costa, David M. Ribeiro, Diogo Coelho, Paula A. Lopes, André M. Almeida, João P. B. Freire, José A. M. Prates

**Affiliations:** 1 CIISA—Centre for Interdisciplinary Research in Animal Health, Faculdade de Medicina Veterinária, Universidade de Lisboa, 1300-477 Lisbon, Portugal; catiamartins@isa.ulisboa.pt (C.F.M.); jpestana@fmv.ulisboa.pt (J.M.P.); cpmateus@fmv.ulisboa.pt (C.M.A.); monicacosta@fmv.ulisboa.pt (M.C.); diogocoelho@fmv.ulisboa.pt (D.C.); ampalopes@fmv.ulisboa.pt (P.A.L.); 2 LEAF—Linking Landscape, Environment, Agriculture and Food, Instituto Superior de Agronomia, Universidade de Lisboa, 1349-017 Lisbon, Portugal; davidribeiro@isa.ulisboa.pt (D.M.R.); aalmeida@isa.ulisboa.pt (A.M.A.); jpfreire@isa.ulisboa.pt (J.P.B.F.)

**Keywords:** *Chlorella vulgaris*, feed enzymes, weaned piglets, meat quality, nutritional quality

## Abstract

*Chlorella vulgaris* (CH) is usually considered a feed supplement in pig nutrition, and its use as an ingredient is poorly studied. Among many interesting characteristics, this microalga has high protein levels and can be a putative alternative for soybean meal. Our aim was to study the effect of a 5% CH incorporation in the diet, individually or combined with two carbohydrases, on meat quality traits and nutritional value. Forty-four post-weaned male piglets individually housed, with an initial live weight of 11.2 ± 0.46 kg, were randomly distributed into four experimental groups: control (*n* = 11, without CH) and three groups fed with 5% CH incorporation, plain (*n* = 10), with 0.005% Rovabio^®^ Excel AP (*n* = 10), and with 0.01% of a pre-selected four-CAZyme mixture (*n* = 11). After two weeks of trial, piglets were slaughtered and *longissimus lumborum* collected. CH had no effect on piglets’ growth performance. In turn, incorporation of CH improved the nutritional value of meat by increasing total carotenoids and *n*-3 PUFA content, thus contributing to a more positive *n*-6/*n*-3 fatty acid ratio. The supplementation with Rovabio^®^ benefited tenderness and increased overall acceptability of pork. Our results show beyond doubt the viability of the utilization of this microalga as a feed ingredient for swine production.

## 1. Introduction

The global population is expected to grow close to approximately 10 billion by 2050, increasing agricultural demand by 50 percent when compared with 2013 [1]. In addition, the growth in income per capita in low- and medium-income countries, and the consequent higher consumption of meat, fruits, and vegetables, will lead to an increase in the land used for agriculture and animal production, with the consequent pressure on natural resources and ecosystems [1]. Among meats, pork is consumed worldwide (36% of total), with a tendency to increase [2]. The sustainability of monogastric production systems depends, therefore, on the suitability of substitute ingredients to corn and soybean. These crops are considered as the basis of monogastric (poultry and swine) feeding. Indeed, there are numerous issues regarding the sustainability of the feedstuffs, given the fact that they are mostly produced in North and South America and transported to consumer markets, with high economic and environmental costs. Furthermore, they are in direct competition with human nutrition [3].

Microalgae have been studied for several economic applications, including animal feeding [4]. Microalgae can be produced in non-agricultural lands. They are photosynthetic organisms able to efficiently transform atmospheric carbon dioxide into high-value products, including carbohydrates, lipids, proteins, and pigments. Therefore, they have promising applications in the food and feed industries [5]. Large-scale cultivation systems and new technologies are currently being developed to turn microalgae cultivation economically feasible [6]. In addition to this challenge, the microalgae cell wall is indigestible by monogastrics. The use of feed enzymes—namely, carbohydrate-active enzyme (CAZymes) that lysate their recalcitrant cell walls—has been demonstrated to be very efficient in improving the nutrient utilization of microalgae by monogastrics [7]. Rovabio^®^ Excel AP is a commercially available CAZyme mixture containing mainly xylanases and β-glucanases for cereal-based diets. This CAZyme mixture has also been used for microalgae-containing diets [8,9]. Moreover, a four-CAZyme mixture, consisting of alginate lyase, exo-β-glucosaminidase, lysozyme, and peptidoglycan *N*-acetylmuramic acid deacetylase, has been shown to partially disrupt the *C. vulgaris* cell wall in vitro [10].

In piglets, weaning is a stressful event derived from social, environmental, and nutritional transitions. In order to decrease the use of antibiotics used to mitigate the piglet post-weaning stress, prebiotics can be a solution. The prebiotic properties of microalgae, in particular the *n*-3 PUFA content, have been studied by different authors [11,12,13]. For instance, *n*-3 PUFA of microalgae improve the fatty acid composition of animal edible tissues, with recognized beneficial health consequences for both humans and animals.

In addition, spit-roasted piglet is a meat that is consumed worldwide, very popular in Mediterranean Europe, Latin America, Louisiana (USA), China, and several islands of Indonesia and the Pacific. It is particularly consumed on special occasions and at family celebrations, such as Christmas. In Mediterranean Europe, it is a highly valued gourmet food, often considered as a regional specialty. For instance, in Portugal the most popular specialities are *Leitão da Bairrada* and *Leitão de Negrais*, whereas in Spain the *Cochinillo Asado* is a reputed specialty of the Castilla-León region. Finally, body composition at the end of post-weaning determines production performance at the growing-finishing period and body composition when pigs achieve 100 kg of body weight [14,15].

This work aimed to study the dietary incorporation of 5% of *C. vulgaris*, with or without exogenous enzymes, on meat quality characteristics and nutritional significance of piglets. We assessed pH, color, lipid oxidation, sensorial qualities, fatty acid composition, and pigment profile. We hypothesized that *C. vulgaris* can be a viable ingredient in piglet feeding by improving the digestibility of valuable microalga nutrients without negatively affecting animal performance and meat traits.

## 2. Materials and Methods

### 2.1. Animals and Experimental Diets

The animal trial was performed at ISA—Instituto Superior de Agronomia (University of Lisbon, Lisbon, Portugal) facilities. All the procedures were reviewed by the Ethics Commission of ISA and accepted by the Animal Care Committee of the National Veterinary Authority (Direção Geral de Alimentação e Veterinária, Lisbon, Portugal), in accordance with the European Union legislation (2010/63/EU Directive). We selected forty-four castrated male piglets from Large White × Landrace sows crossed with Pietrain boars; they were weaned at 28 days of age and had an initial body weight of 11.2 ± 0.46 kg (mean ± SD). The piglets were single housed in metabolic cages (1000 × 500 × 480 mm). During the adaptation period of two days, to minimize stress and stabilize all metabolic conditions, two animals failed to adapt and were not considered in the experiment. The remaining piglets were arbitrarily distributed in 4 experimental groups: Control (*n* = 11, cereal and soybean meal-based diet), CH (*n* = 10, control diet with 5% *C. vulgaris*), CH + R (*n* = 10, control diet with 5% *C. vulgaris* and 0.005% Rovabio^®^ Excel AP from Adisseo (Antony, France)), and CH + M (*n* = 11, control diet with 5% *C. vulgaris* supplemented and 0.01% of a pre-selected four-CAZyme mixture (previously described by Coelho et al. [10])). *C. vulgaris* was produced as described in detail by Coelho et al. [10]. Then, this microalga was supplied as freeze-dried powder (Allmicroalgae—Natural Products SA, Pataias, Portugal) and incorporated in the diets. Rovabio^®^ Excel AP was incorporated in the diet at a 0.005% level following the manufacturer’s recommendation.

Diets were dried at 103 °C to constant weight to assess dry matter (DM). Crude protein of diets was determined following the method 954.01 [16] utilizing the factor 6.25 × nitrogen content (N) calculated by the Kjeldahl procedure. Crude fat of diets was assessed by an automatic Soxhlet extraction with petroleum ether (Gerhardt Analytical Systems, Königswinter, Germany). Ash content of the experimental diets was assessed following the 942.05 [16] method. Neutral detergent fiber (NDF) and acid detergent fiber (ADF) were determined by 989.03 [16] method. Metabolizable energy (ME) was estimated in accordance with Noblet et al. [17]. Fatty acids were determined by one-step extraction and converted to fatty acid methyl esters (FAME) through acid transesterification and gas chromatography (GC) having heneicosanoic acid (21:0) methyl ester as the internal standard [18]. β-Carotene and tocopherols of diets were determined by direct saponification, with a single *n*-hexane extraction followed by HPLC, based on the external standard technique from a standard curve of peak area vs. concentration, as previously reported [19]. The determination of pigments in diets was carried out in accordance with Teimouri et al. [20], with minor alterations. In brief, diets (0.5 g) were incubated at room temperature with acetone overnight under agitation and in the dark. Following on extraction, samples were subjected to centrifugation at 4000 rpm during 5 min and analyzed by UV/Vis spectrophotometry (Ultrospec 3100; Amersham Biosciences, Little Chalfont, UK). The concentration of pigments was assessed using methodologies described by Hynstova et al. [21] equations. All diets were formulated to have 3440 kcal ME/kg of energy and 19.5% of crude protein, as fed basis. The ingredients and chemical composition of diets are shown in Table 1. The detailed chemical composition of *C. vulgaris* was previously described [9].

### 2.2. Productive Parameters

Throughout the animal trial, feed and refusals were recorded daily. Animals were weighed once a week before feeding to calculate average daily feed intake (ADFI), average daily weight gain (ADG), and feed conversion ratio (FCR). After 15 days of the experiment, piglets were slaughtered at a body weight of 23.1 ± 2.56 kg, through electrical stunning followed by exsanguination, in accordance with standard protocols applied in commercial abattoirs. *Longissimus lumborum* muscle samples were extracted from both sides of the carcass, between the third and fifth lumbar vertebrae. Muscle samples from the left carcass side were collected, minced, vacuum packed, and stored at −20 °C for intramuscular fat and fatty acid profile and for total pigments and tocopherol profile determinations. For TBARS analysis, muscles samples were stored at −80 °C. Muscle samples from the right carcass side were stored at 4 °C during 24 h for color and pH determinations. Then, the samples were vacuum packed and frozen at −20 °C until cooking loss, shear force, and sensory analyses.

### 2.3. Determination of Meat Quality Traits

The pH of *longissimus lumborum* at 24 h postmortem was measured using a pH meter with a glass penetrating electrode from Hanna Instruments (Woonsocket, RI, USA) and was determined as an average of 3 replicates. Meat color variables, such as lightness (L*), redness (a*), and yellowness (b*) were measured 24 h postmortem on 3 spots of cut surface of the *longissimus lumborum* samples using a colorimeter (Minolta CR-300; Konica Minolta, Tokyo, Japan) after 1 h at 4 °C. Lipid oxidation of meat was assessed by thiobarbituric acid reactive substances (TBARS) at days 0 and 8, stored at 4 °C, following the procedure of Grau et al. [22]. TBARS were calculated in duplicate from a standard curve of 1,1,3,3-tetraethoxypropane (Fluka, Neu Ulm, Germany) and expressed as mg of malondialdehyde/kg of muscle.

### 2.4. Determination of Cooking Loss and Shear Force

Meat samples were thawed at 4 °C overnight and cooked using a water bath at 80 °C until reaching 78 °C of internal temperature, monitored by a thermocouple (Lufft C120; Lufft, München, Germany). After 2 h cooling at room temperature, samples were longitudinally cut toward the fibers with a 1 cm^2^ cross-section for cooking loss and shear force. Before and after cooking, meat samples were weighed to determine cooking loss. Meat shear force was determined using a Warner-Bratzler blade coupled to a texture analyzer (TA-XT Plus texture analyzer; Stable Micro Systems, Surrey, UK) and is expressed as the mean of the peak value of a minimum of 4 replicate measurements.

### 2.5. Trained Sensory Panel Analysis

Trained sensory analysis was carried out in muscle samples, trimmed of external connective tissue, cut into cubes with approximately 1 cm^3^, and cooked in a water bath, as previously mentioned for cooking loss. Samples were arbitrarily allocated across 5 panel sessions, with 8 random samples per session. The attributes were tenderness, juiciness, flavor, off-flavor, and overall acceptability in a numeric scale from 1 to 8, in which 1 was the low/negative score and 8 was the high/positive score. For off-flavor, the scale applied was from 0 (absence) to 8 (maximum). The sensory panel consisted of thirteen panelists, selected after intensive training, according to Cross et al. [23].

### 2.6. Determination of Intramuscular Fat and Fatty Acid Profile

Intramuscular fat from lyophilized *longissimus lumborum* samples was extracted according to Folch et al. [24], utilizing dichloromethane–methanol (2:1, *v*/*v*) as reported by Carlson [25], and measured gravimetrically by weighing the fatty residue after solvent evaporation. Fatty acids were converted to FAME through a combined alkaline and acid sequential transesterification, in accordance with Raes et al. [26]. The fatty acid composition was analyzed by GC (HP6890A; Hewlett-Packard, Avondale, PA, USA), equipped with a flame ionization detector, as described [9]. The identification of FAME was achieved using a reference standard (FAME mixture of 37 compounds, Supelco Inc., Bellefonte, PA, USA) corroborated by GC along with mass spectrometry using a GC-MS QP2010-Plus (Shimadzu, Kyoto, Japan). FAME calculation was based on the internal standard technique with heneicosanoic acid (21:0). Fatty acids are expressed as a percentage of the sum of identified fatty acids.

### 2.7. Determination of Total Pigments, Cholesterol, and Tocopherols

Chlorophyll a, chlorophyll b, and total carotenoids contents were quantified in meat, in accordance with Teimouri et al. [20]. Samples were subjected to incubation overnight with acetone (Merck KGaA, Darmstadt, Germany) and agitation at room temperature in the dark. Following on centrifugation, the absorbance was read at a UV/Vis spectrophotometer (Ultrospec 3100 pro; Amersham Biosciences, Little Chalfont, UK) and results were determined in accordance with Hynstova et al. [21]. The parallel quantification of total cholesterol, β-Carotene, and tocopherols, in duplicate, in meat samples was carried out, according to Prates et al. [19].

### 2.8. Statistics

All data were analyzed with the PROC GLM of SAS software package (version 9.4; SAS Institute Inc., Cary, NC, USA). Data were checked for normal distribution and variance homogeneity. The statistical model assumed the dietary treatment as the single effect and the piglet as the experimental unit. When significant effects of dietary treatments were observed, least-squares means for multiple comparisons were generated using the PDIFF option adjusted with Tukey–Kramer method. Results were considered significantly different at *p* < 0.05.

## 3. Results

### 3.1. Zootechnical Parameters

Table 2 shows results on growth performance parameters and feed intake of piglets. Diets had no significant effect on growth performance variables, such as final live weight, ADG, and FCR (*p* > 0.05). The reference group had lower ADFI than groups fed with *C. vulgaris* (*p* = 0.008), although this difference had no impact on piglets’ growth.

### 3.2. Meat Quality Traits

#### 3.2.1. pH, Color, and Susceptibility to Lipid Oxidation

The impact of experimental diets on meat quality traits from piglets is shown in Table 3. Diets did not affect pH 24 h postmortem and color parameters (*p* > 0.05). Although TBARS were not detected in meat at day 0, their levels were diminished in the reference group relative to CH + M (0.151 vs. 0.805 mg of malondialdehyde/kg of muscle, respectively) after 8 days under refrigeration (*p* = 0.019).

#### 3.2.2. Cooking Loss, Shear Force, and Sensory Panel Analysis

Table 4 shows the impact of experimental diets on cooking loss, shear force, and sensory panel analysis of meat. Cooking loss had a statistically higher value in the control group compared with the CH + M group (*p* = 0.011). Shear force was unaffected by diets (*p* > 0.05). Juiciness, flavor, and off-flavor presented no significant differences among diets (*p* > 0.05). However, for tenderness, the CH + R group showed the tenderest meat (*p* < 0.001). In line with this finding, the overall acceptability was higher in the CH + R muscle compared with the other diets (*p* = 0.000).

### 3.3. Intramuscular Fat, Total Cholesterol, and Fatty Acid Profile of Meat

The impact of experimental diets on intramuscular fat, total cholesterol, and fatty acid profile of *longissimus lumborum* muscle samples is shown in Table 5. Intramuscular fat and cholesterol contents were unaffected by diets (*p* > 0.05). Dietary treatments influenced only a few fatty acids, specifically 15:0, 17:0, 17:1*n-8*, 18:0, 20:1*n-9*, 20:2*n*-6, 22:1*n*-9, 22:5*n*-3, and 22:6*n*-3. Compared with Chlorella-fed piglets, the control group had a higher percentage of 15:0 (*p* < 0.0001), 17:0 (*p* < 0.001), and 17:1*n-8* (*p* < 0.001). Interestingly, stearic acid (18:0) was lower in control and CH + M groups in comparison with the other groups (*p* = 0.023). In contrast, the proportion of 20:2*n*-6 increased in piglets fed control and CH + M diets (*p* = 0.004). Additionally, the reference group had a higher proportion of 20:1*n-9* (*p* = 0.009) but a lower proportion of 22:1*n*-9 (*p* = 0.004) than piglets fed CH. Conversely, the proportions of 22:5*n*-3 (DPA, docosapentaenoic acid) (*p* < 0.001) and 22:6*n*-3 (DHA, docosahexaenoic acid) (*p* = 0.001) were enhanced in piglets fed CH and CH + M. Indeed, both DPA and DHA increased at least 1.79-fold and 2.35-fold in CH and CH + M groups, respectively.

Concerning the partial sums and ratios of fatty acids, only the *n*-3 PUFA sum was enhanced in CH and CH + M groups (*p* < 0.001) compared with the other groups. The remaining partial sums of fatty acids, as well as the PUFA/SFA ratio, were similar across all dietary treatments (*p* > 0.05). Nevertheless, the *n*-6/*n*-3 ratio was reduced in microalga-fed groups in comparison with the control group (*p* < 0.001).

### 3.4. Total Pigments and Tocopherol Profile of Meat

Total carotenoids, chlorophylls, and tocopherols of *longissimus lumborum* samples are shown in Table 6. The pigment contents and tocopherol profile were identical for all dietary treatments (*p* > 0.05), expect for total carotenoids. Meat from Chlorella-fed piglets had values of total carotenoids 2 times higher than the reference group (*p* = 0.002). β-Carotene was undetected in any of the groups.

## 4. Discussion

To the best of our knowledge, this is the first study ever to use *C. vulgaris* microalga as a feedstuff in piglets’ diet, supplemented or not with exogenous enzyme cocktails, such as the Rovabio^®^ Excel AP and the preselected four-CAZyme mixture [10]. In this work, a zootechnical trial was performed along with the determination of pork quality and nutritional traits. The dietary incorporation of 5% of *C. vulgaris* had no impact on growth performance of piglets. In agreement, Furbeyre et al. [11] using Spirulina and *C. vulgaris*, both at a supplement level of 1%, showed no effects over ADFI and ADG in weaned piglets (9.1 to 20 kg LW). The authors studied the administration of the same microalgae via drinking water (385 mg/kg LW) and found no effect on growth performance in suckling (4.9 kg LW) and weaned piglets (9.04 kg LW) [11]. Like other studies using microalgae as a dietary supplement, Yan et al. [27] described that 0.1 and 0.2% dietary incorporation of fermented *C. vulgaris* in pigs’ diets (26.6 to 53.0 kg LW) promoted an increase in the ADG of 655 g/d relative to the reference diet. For the first time, Martins et al. [8] used Spirulina as an ingredient (10% of dietary inclusion) and described that the growth performance of piglets was reduced, thus highlighting the need of feed enzymes to enhance the digestive utilization of this microalga. In our study, no significant effects on the growth performance of piglets were found, revealing that the dietary level of 5% *C. vulgaris* did not compromise the productive variables. The exogenous carbohydrases applied had no consequences to the point of a higher level of supplementation being necessary, as advanced by Martins et al. [8].

Regarding meat quality traits, the level of 5% *C. vulgaris* incorporation*,* when combined with the pre-selected four-CAZyme mixture, only affected the oxidative stability of *longissimus lumborum* at day 8 postmortem (storage at 4 °C). After 8 days under refrigeration, the increased TBARS reflect a higher instability of meat from microalga-fed piglets with the four-CAZyme mixture in comparison with the control group. This is likely due to poor radical-scavenging activity of the intrinsic antioxidants for mitigating the lipid oxidation promoted by enhanced *n*-3 PUFA content. TBARS over 0.5 mg malondialdehyde/kg of fresh meat are recognized as crucial since, at this level of lipid oxidation, the rancid off-flavors are easily perceived by the consumers [28]. In the current study, only at day 8 of storage, TBARS were above this threshold value. Moreover, TBARS values for the four-CAZyme mixture diet-fed animals were lower than 0.9 mg malondialdehyde/kg of meat, proposed by Jayasingh and Cornforth [29] for ground and cooked pork. Martins et al. [8] found that in comparison with the reference diet, the incorporation of 10% of Spirulina in piglets’ diet, without enzyme supplementation, increased TBARS at three days of storage under refrigeration. Likewise, data on the oxidative stability of meat did not match the antioxidant power of Spirulina, as in the present case of *C. vulgaris*.

An existing relationship between cooking loss and juiciness in pork was described by Aaslyng et al. [30]. The higher value in cooking loss found in the reference group influenced the lower value of juiciness for the same diet. Sensory attributes such as tenderness and overall acceptability were increased by Rovabio^®^ commercial supplementation relative to the other diets, suggesting that overall consumer acceptability is mostly determined by tenderness. Furthermore, and according to our trained sensory panel, *C. vulgaris* had no negative effect on meat flavor, thus contributing to consumer’s acceptance of this meat.

Feeding piglets with 5% of *C. vulgaris*, individually or combined with the four-CAZyme mixture, increased DPA and DHA, showing a positive correspondence between *n*-3 PUFA in the diet and *n*-3 PUFA deposited in *longissimus lumborum* muscle. *n*-3 long-chain PUFA display health beneficial effects [31]. In fact, several animal and epidemiological reports have proven the advantages of *n*-3 PUFA on cardiovascular disease outcomes [32,33]. Furthermore, the FAO, the WHO, and the American Heart Association recommended EPA (20:5*n*-3; eicosapentaenoic acid) plus DHA daily intake from 140 to 600 mg/d, depending on the authority guidelines [34,35]. However, most Western populations consume an average below 500 mg/day of *n*-3 long-chain PUFA [36]. For instance, piglets’ diet receiving 5% of *C. vulgaris* combined with the four-CAZyme mixture could be a valuable source of these protective fatty acids to both animals and humans. Consistent with our findings, the dietary *C. vulgaris* at this level of incorporation also produced an increment in *n*-3 PUFA amount in finishing pigs [9]. The enhancement of *n*-3 PUFA content subsequently resulted in a positive decline in *n*-6/*n*-3 ratio in muscle with incorporation of *C. vulgaris* in piglets’ diet. Although the *n*-6/*n*-3 ratios were considerably elevated, our data indicate that meat from piglets fed this microalga complies more (around 12.6) with the advised *n*-6/*n*-3 ratio (below 4), thus promoting health-protecting cardiovascular effects for consumers [37] and improving meat quality.

A significant increase of total carotenoids in *longissimus lumborum* muscle was observed in piglets fed *C. vulgaris*, which reflects diet composition. In fact, the incorporation of this microalga led to higher content of pigments in the diets, in particular about 17 times more total carotenoids if compared with the reference diet. As highlighted by Coelho et al. [9], the transfer of carotenoids from the microalga to the meat adds extra nutritional value to pork. Our data are in accordance with these authors, who also found 2 times higher total carotenoid contents in meat from finishing pigs fed with 5% of *C. vulgaris*. Similar to the study by Coelho et al. [9], β-Carotene (pro-vitamin A) was undetected in meat, possibly indicating that this pigment was rapidly metabolized into vitamin A because pigs are unable to synthesize carotenoids.

## 5. Conclusions

The incorporation of *C. vulgaris* at a level of 5% in the diet does not impair growth performance of piglets or their meat quality traits. In contrast, at this level of dietary inclusion, it seems that an improvement in the nutritional value of pork occurs, in particular through the increment of total carotenoids and *n*-3 PUFA content, which promotes a beneficial *n*-6/*n*-3 PUFA ratio for the consumers. Additionally, the supplementation with exogenous enzymes, both the commercial Rovabio^®^ formulation and the pre-selected four-CAZyme mixture, seems to have a minor impact on the multiple parameters assessed. One exception is the increased score for tenderness and overall acceptability of pork from piglets fed *C. vulgaris* combined with Rovabio^®^. In view of these findings, further research is warranted, focusing in particular on higher levels of *C. vulgaris* incorporation, individually or supplemented with feed enzymes, in order to ascertain whether *C. vulgaris* is a cost-effective alternative feedstock for livestock production.

## Figures and Tables

**Table 1:** Ingredients and chemical composition of experimental diets.

	Diets			
Item	Control	CH	CH + R	CH + M
**Ingredients (% as fed basis)**				
Wheat	43.9	44.0	44.0	44.0
Corn	15.0	15.0	15.0	15.0
Soybean meal 48	25.0	20.0	20.0	20.0
Whey powder	10.0	10.0	10.0	10.0
Soybean oil	3.00	3.00	3.00	3.00
*Chlorella vulgaris*	0	5.00	5.00	5.00
Rovabio^®^ Excel AP	0	0	0.005	0
Four-CAZyme mixture	0	0	0	0.01
**Energy (kcal ME ^1^/kg as fed basis)**	3428	3436	3449	3449
**Proximate composition (% as fed basis)**				
Dry matter	90.5	90.8	90.8	90.9
Crude protein	19.3	19.2	19.5	19.4
Crude fat	5.29	5.39	5.39	5.63
Ash	5.43	5.65	5.47	5.60
NDF	12.9	11.9	12.9	10.4
ADF	2.76	2.45	2.58	2.54
**Fatty acid composition (% total fatty acids)**				
14:0	0.351	0.380	0.380	0.361
16:0	10.6	11.0	10.9	11.1
16:1*n-7*	0.158	0.903	0.900	0.677
17:0	0.095	0.104	0.103	0.104
17:1*n-8*	0.040	0.583	0.643	0.828
18:0	3.35	3.33	3.38	3.26
18:1*n-9*	24.8	24.5	24.5	24.6
18:1*n-7*	0.909	1.16	1.16	1.10
18:2*n*-6	55.8	53.3	53.2	52.9
18:3*n*-3	1.55	2.18	2.23	2.52
**β-Carotene and tocopherol profile (μg/g)**				
β-Carotene	n.d.	13.3	13.7	14.5
α-Tocopherol	28.6	19.9	22.1	24.2
β-Tocopherol	1.11	1.10	1.00	1.12
γ-Tocopherol	2.52	2.00	2.21	2.11
δ-Tocopherol	0.502	0.334	0.387	0.396
α-Tocotrienol	3.43	3.73	3.58	3.53
γ-Tocotrienol	1.38	1.51	1.69	1.46
**Pigments (µg/g)**				
Chlorophyll-a ^2^	3.38	109	130	135
Chlorophyll-b ^3^	6.05	31.9	42.6	39.8
Total chlorophylls ^4^	9.43	141	172	174
Total carotenoids ^5^	2.67	36.9	44.5	52.9
Total chlorophylls and total carotenoids ^6^	12.1	178	217	227

Dietary treatments: CH, *Chlorella vulgaris* diet; CH + R, *C. vulgaris* diet supplemented with 0.005% of Rovabio^®^ Excel AP; CH + M, *C. vulgaris* diet supplemented with 0.01% of four-CAZyme mixture. ADF, acid detergent fiber; NDF, neutral detergent fiber; ME, metabolizable energy; n.d., not detected. ^1^ Metabolizable energy (kcal/kg DM) = 4412 − 11.06 × ash (g/kg DM) + 3.37 × crude fat (g/kg DM) − 5.18 × ADF (g/kg DM). ^2^ Ca = 11.24 A_662_ − 2.04 A_645_. ^3^ Cb = 20.13 A_645_ − 4.19 A_662_. ^4^ Ca + b = 7.05 A_662_ + 18.09 A_645_. ^5^ Cx + c = (1000 A_470_ − 1.90 Ca−63.14 Cb)/214. ^6^ (Ca + b) + (Cx + c). Bolded words thorought the table are topic headings.

**Table 2:** Effect of experimental diets on feed intake and growth performance of piglets.

	Diets					
	Control	CH	CH + R	CH + M	SEM	*p*-Value
**Initial live weight (kg)**	11.1	11.1	11.3	11.2	0.105	0.851
**Final live weight (kg)**	22.3	23.3	23.5	23.1	0.247	0.349
**ADFI (g) ^1^**	768 ^a^	852 ^b^	857 ^b^	856 ^b^	11.6	0.008
**ADG (g) ^2^**	535	581	579	569	8.50	0.189
**FCR ^3^**	1.44	1.47	1.48	1.51	0.013	0.282

Dietary treatments: CH, *Chlorella vulgaris* diet; CH + R, *C. vulgaris* diet supplemented with 0.005% of Rovabio^®^ Excel AP; CH + M, *C. vulgaris* diet supplemented with 0.01% of four-CAZyme mixture. ^1^ ADFI, average daily feed intake. ^2^ ADG, average daily weight gain. ^3^ FCR, feed conversion ratio. ^a,b^ Values with different superscript letters in the same row are significantly different (*p* < 0.05). Bolded words thorought the table are topic headings.

**Table 3:** Effect of experimental diets on pH 24 h, CIE color parameters (L*, a*, b*) and TBARS levels (mg malondialdehyde/kg muscle) after 0 and 8 days under refrigeration in *longissimus lumborum* muscle.

	Diets					
	Control	CH	CH + R	CH + M	SEM	*p*-Value
**pH 24 h**	5.61	5.54	5.57	5.61	0.033	0.401
**Color**						
L*	48.6	48.6	48.5	47.3	0.810	0.582
a*	6.20	6.50	6.79	7.26	0.321	0.112
b*	−0.528	−0.157	−0.391	−0.821	0.2458	0.275
**TBARS ^1^**						
Day 0	n.d.	n.d.	n.d.	n.d.	-	-
Day 8	0.151 ^a^	0.752 ^ab^	0.621 ^ab^	0.805 ^b^	0.161	0.019

Dietary treatments: CH, *Chlorella vulgaris* diet; CH + R, *C. vulgaris* diet supplemented with 0.005% of Rovabio^®^ Excel AP; CH + M, *C. vulgaris* diet supplemented with 0.01% of four-CAZyme mixture. ^1^ TBARS-thiobarbituric acid reactive substances; n.d., not detected (<0.020 mg malondialdehyde/kg muscle). ^a,b^ Values with different superscript letters in the same row are significantly different (*p* < 0.05). Bolded words thorought the table are topic headings.

**Table 4:** Effect of experimental diets on cooking loss (%), shear force (kg) and sensory panel analysis in *longissimus lumborum*.

	Diets					
	Control	CH	CH + R	CH + M	SEM	*p*-Value
**Cooking loss**	28.5 ^b^	27.5 ^ab^	25.5 ^ab^	24.7 ^a^	0.881	0.011
**Shear force**	3.99	4.04	3.68	4.29	0.269	0.466
**Sensory panel scores**						
Tenderness	4.95 ^a^	5.10 ^a^	5.69 ^b^	5.04 ^a^	0.121	<0.001
Juiciness	5.08	5.34	5.48	5.21	0.109	0.056
Flavor	4.90	4.92	4.96	4.96	0.111	0.965
Off-flavor	0.276	0.362	0.298	0.378	0.0764	0.714
Overall acceptability	4.91 ^a^	5.09 ^a^	5.54 ^b^	5.03 ^a^	0.112	0.000

Dietary treatments: CH, *Chlorella vulgaris* diet; CH + R, *C. vulgaris* diet supplemented with 0.005% of Rovabio^®^ Excel AP; CH + M, *C. vulgaris* diet supplemented with 0.01% of four-CAZyme mixture. ^a,b^ Values with different superscript letters in the same row are significantly different (*p* < 0.05). Bolded words thorought the table are topic headings.

**Table 5:** Effect of experimental diets on intramuscular fat content (g/100 g muscle), total cholesterol (mg/100 g muscle), and fatty acid (FA) composition (% of total FA) in *longissimus lumborum*.

	Diets					
	Control	CH	CH + R	CH + M	SEM	*p*-Value
**Intramuscular fat**	1.36	1.14	1.31	1.35	0.078	0.159
**Total cholesterol**	54.8	53.4	44.4	48.4	0.029	0.058
**Fatty acid composition**						
10:0	0.024	0.028	0.032	0.019	0.005	0.204
12:0	0.046	0.049	0.047	0.054	0.005	0.660
14:0	0.839	0.826	0.943	0.948	0.091	0.661
14:1*n-5*	0.015	0.015	0.015	0.012	0.003	0.924
15:0	0.310 ^b^	0.149 ^a^	0.128 ^a^	0.135 ^a^	0.028	<0.001
DMA-16:0	0.128	0.131	0.122	0.136	0.022	0.974
16:0	21.7	23.8	24.5	23.7	1.03	0.244
16:1*n-9*	0.442	0.510	0.468	0.487	0.017	0.043
16:1*n-7*	2.11	1.95	2.51	2.24	0.170	0.139
17:0	1.25 ^b^	0.730 ^a^	0.620 ^a^	0.710 ^a^	0.122	0.001
17:1*n-8*	0.864 ^b^	0.500 ^a^	0.451 ^a^	0.467 ^a^	0.080	0.001
DMA-18:0	0.030	0.039	0.027	0.043	0.006	0.210
DMA-18:1	0.029	0.053	0.036	0.047	0.009	0.203
18:0	12.7 ^a^	14.3 ^b^	14.3 ^b^	13.8 ^ab^	0.427	0.023
18:1*n-9*	28.8	26.8	30.1	29.0	1.26	0.314
18:1*n-7*	3.35	3.31	3.25	3.26	0.073	0.717
18:2*n*-6	19.3	17.9	15.9	16.7	1.85	0.558
18:2*t*9*t*12	0.074	0.070	0.074	0.073	0.007	0.979
18:3*n*-6	0.131	0.149	0.106	0.113	0.020	0.431
18:3*n*-3	0.382	0.358	0.378	0.424	0.050	0.809
20:0	0.187	0.210	0.187	0.190	0.010	0.333
20:1*n-9*	0.586 ^b^	0.463 ^a^	0.543 ^ab^	0.514 ^ab^	0.025	0.009
20:2*n*-6	0.720 ^b^	0.487 ^a^	0.465 ^a^	0.514 ^ab^	0.053	0.004
20:3*n*-6	0.369	0.358	0.251	0.295	0.054	0.363
20:4*n*-6	2.69	3.07	1.64	2.11	0.462	0.150
20:3*n*-3	0.056	0.051	0.057	0.051	0.007	0.868
20:5*n*-3	0.053	0.082	0.060	0.065	0.009	0.139
22:0	0.083	0.110	0.092	0.085	0.009	0.171
22:1*n*-9	0.050 ^a^	0.093 ^b^	0.069 ^ab^	0.065 ^ab^	0.008	0.004
22:2*n*-6	0.040	0.040	0.035	0.032	0.007	0.766
22:5*n*-3	0.275 ^a^	0.595 ^b^	0.231 ^a^	0.491 ^b^	0.056	<0.001
22:6*n*-3	0.305 ^a^	0.889 ^b^	0.621 ^ab^	0.716 ^b^	0.096	0.001
23:0	0.155	0.254	0.181	0.202	0.026	0.062
Others	1.89	1.63	1.56	2.35	0.299	0.229
**Partial sums of fatty acids**						
∑ SFA ^1^	37.3	40.4	41.0	39.8	1.29	0.173
∑ MUFA ^2^	36.2	33.6	37.4	36.0	1.40	0.288
∑ PUFA ^3^	24.4	24.1	19.8	21.6	2.36	0.458
∑ *n*-3 PUFA ^4^	1.07 ^a^	1.97 ^b^	1.35 ^a^	1.75 ^b^	0.101	<0.001
∑ *n*-6 PUFA ^5^	23.3	22.0	18.4	19.8	2.38	0.453
**Ratios of fatty acids**						
PUFA/SFA	0.661	0.632	0.491	0.573	0.075	0.386
*n*-6/*n*-3	21.9 ^b^	11.7 ^a^	13.5 ^a^	12.7 ^a^	1.51	<0.001

Dietary treatments: CH, *Chlorella vulgaris* diet; CH + R, *C. vulgaris* diet supplemented with 0.005% of Rovabio^®^ Excel AP; CH + L, *C. vulgaris* diet supplemented with 0.01% of four-CAZyme mixture. SEM, standard error of the mean; FA, fatty acids; DMA, dimethylacetal; SFA, saturated fatty acids; MUFA, monounsaturated fatty acids; PUFA, polyunsaturated fatty acids. ^1^ 10:0 + 12:0 + 14:0 + 15:0 + 16:0 + 17:0 + 18:0 + 20:0 + 22:0 + 23:0. ^2^ 14:1*n-5* + 16:1*n-9* + 16:1*n-7* + 17:1*n-8* + 18:1*n-9* + 18:1*n-7* + 20:1*n-9* + 22:1*n*-9. ^3^ 18:2*n*-6 + 18:3*n*-6 + 18:3*n*-3 + 20:2*n*-6 + 20:3*n*-6 + 20:4*n*-6 + 20:3*n*-3 + 20:5*n*-3 + 22:2*n*-6 + 22:5*n*-3 + 22:6*n*-3. ^4^ 18:3*n*-3 + 20:3*n*-3 + 20:5*n*-3 + 22:5*n*-3 + 22:6*n*-3. ^5^ 18:2*n*-6 + 18:3*n*-6 + 20:2*n*-6 + 20:3*n*-6 + 20:4*n*-6. ^a,b^ Values with different superscript letters in the same row are significantly different (*p* < 0.05). Bolded words thorought the table are topic headings.

**Table 6:** Effect of experimental diets on total pigments and tocopherol profile (µg/g) in *longissimus lumborum*.

	Diets					
	Control	CH	CH + R	CH + M	SEM	*p*-Value
**Pigments**						
β-Carotene	n.d	n.d	n.d	n.d	-	-
Chlorophyll-a ^1^	6.87	14.1	14.1	16.4	2.92	0.107
Chlorophyll-b ^2^	13.3	22.5	21.9	25.0	5.44	0.420
Total chlorophylls ^3^	20.2	36.5	36.0	41.4	8.30	0.273
Total carotenoids ^4^	3.75 ^a^	7.14 ^b^	7.99 ^b^	7.51 ^b^	0.819	0.002
Total chlorophylls and total carotenoids ^5^	23.9	43.7	44.1	48.9	8.49	0.154
**Tocopherols**						
α-Tocopherol	1.13	1.08	0.947	1.03	0.066	0.257
γ-Tocopherol	0.025	0.024	0.026	0.027	0.001	0.217

Dietary treatments: CH, *Chlorella vulgaris* diet; CH + R, *C. vulgaris* diet supplemented with 0.005% of Rovabio^®^ Excel AP; CH + M, *C. vulgaris* diet supplemented with 0.01% of four-CAZyme mixture. ^1^ Ca = 11.24 A_662_ − 2.04 A_645_. ^2^ Cb = 20.13 A_645_ − 4.19 A_662_. ^3^ Ca + b = 7.05 A_662_ + 18.09 A_645_. ^4^ Cx + c = (1000 A_470_ − 1.90 Ca − 63.14 Cb)/214. ^5^ (Ca + b) + (Cx + c); n.d., not detected. ^a,b^ Values with different superscript letters in the same row are significantly different (*p* < 0.05). Bolded words thorought the table are topic headings.

## Data Availability

The data presented in this study are available on request from the corresponding author.
